# *Neisseria meningitidis* Strain of Unknown Serogroup, China

**DOI:** 10.3201/eid1703.101329

**Published:** 2011-03

**Authors:** Haijian Zhou, Zhujun Shao, Qian Li, Li Xu, Jiang Wu, Biao Kan, Jianguo Xu

**Affiliations:** Author’s affiliation: National Institute for Communicable Disease Control and Prevention, Beijing, People’s Republic of China (H. Zhou, Z. Shao, L. Xu, B. Kan, J. Xu);; State Key Laboratory for Infectious Disease Prevention and Control, Beijing (H. Zhou, Z. Shao, Q. Li, B. Kan, J. Xu);; Beijing Center for Disease Prevention and Control, Beijing (J. Wu)

**Keywords:** Bacteria, Neisseria meningitidis, nonserogroupable, China, bacteria, letter

**To the Editor:** *Neisseria meningitidis* is a major public health hazard in many parts of the world. This organism is classified into 13 serogroups, and most meningococcal disease is caused by strains that express 1 of the 5 types of capsular polysaccharides (A, B, C, Y, and W135). In the natural reservoir of the human nasopharynx, strains of *N. meningitidis* that do not fit into 1 of the 13 serogroups and are presumably unencapsulated are common. By contrast, rare meningococcal diseases are caused by these nonserogroupable strains. In this article, we describe a case of *N. meningitidis* infection caused by a nonserogroupable strain in the People’s Republic of China and the genotype characteristics of this strain.

The patient was a 6-month-old boy who was admitted to a local hospital in Beijing in May 2009. The infection started suddenly with high fever (39°C). *N. meningitidis* infection was confirmed on the basis of the clinical signs and results of laboratory examination. Nausea, vomiting, and neck stiffness developed, and the patient lost consciousness. Physical examination showed a positive Kernig sign and negative Brudzinski sign. The patient’s cerebrospinal fluid sample was injected into chocolate agar, in which microbial growth was observed after 24 hours. The API NH system (bioMérieux, Marcy-Etoile, France) showed that the isolate was *N. meningitidis*. However, this strain could not be placed in a serogroup, even after specific antiserum (Remel, Lenexa, KS, USA) was used. No other disease with complement deficiency was detected in the patient. The patient’s infection was treated with antimicrobial drugs, and he recovered completely.

We investigated this nonserogroupable *N. meningitidis* strain by multilocus sequence typing (MLST), pulsed-field gel electrophoresis (PFGE), and subtyping of the variable regions of the genes (*por*A, *por*B, and *fet*A) encoding the outer membrane proteins. MLST indicated that the *fum*C was a new allele with a new number 482. The allele numbers for *abc*Z, *adk, fum*C, *gdh, pdh*C, and *pgm* were 222, 3, 58, 386, 18, and 77, respectively. This strain was assigned a new sequence type number, ST7962.

Among the 44 complexes designed in the MLST database, ST7962 was most similar to the ST4821 complex with 3 identical loci. The PFGE pattern of this strain was compared with the PFGE patterns in the reference database of *N. meningitidis* from China by using BioNumerics version 5.10 software (Applied Maths, Kortrijk, Belgium). At the time of comparison, the database contained 618 isolates of *N. meningitidis* and 243 PFGE patterns. This strain had a single pattern that was clustered together with the ST4821 complex strains in the cluster tree based on the PFGE patterns. The PorA genotype of the strain was determined to be P1.7–2, 14, which was a genotype associated with the ST4821 complex serogroup C strains that caused outbreaks in China in 2003 (*1*). The *por*B and *fet*A alleles of this strain were 3–18 and F4–21, respectively.

The genetic basis for the reason that this strain was nonserogroupable was studied by PCR and sequencing. PCR showed that this strain had intact capsule genetic islands of *ctr*A-D, *syn*A-C, *lip*A, and *lip*B, and contained *syn*D, encoding the serogroup B polysialyltransferase. The capsular gene clusters were sequenced entirely, and a missense mutation within *syn*D was identified (Figure). The mechanism underlying the capsule phase variation of *N. meningitidis* serogroup B involves a variation caused by slipped-strand mispairing in the polyC tract at the 5′ end of *syn*D (*2**–**4*). A tract of 7 C residues encodes capsular expression, and an insertion or deletion of 1 C results in a missense mutation within *syn*D, thereby leading to nonexpression of the capsule. Nucleotide sequencing of *syn*D of our isolate revealed an insertion of 1 C within the polyC tract. Thus, slipped-strand mispairing within *syn*D was predicted to be the mechanism underlying the nonserogroupability of this strain.

**Figure:**
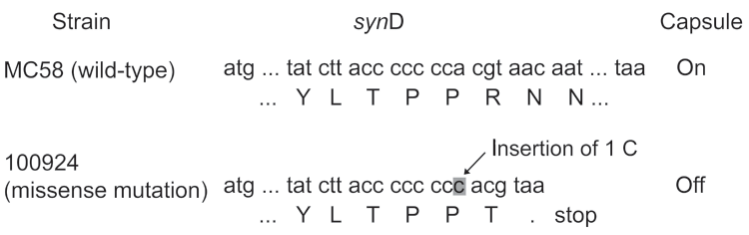
Genetic basis for the *Neisseria meningitidis* strain that cannot be placed in a known serogroup. A predicted slipped-strand mispairing occurred within *synD*, which encodes the serogroup B sialyltransferase. In wild-type *N. meningitidis* serogroup B (MC58), the *syn*D polyC tract contains 7 C residues, and capsule is expressed. When an insertion (as in isolate 100924) of 1 C residue occurs, a result of local denaturation and mispairing followed by replication or repair, a premature stop codon is generated, and the capsule is not expressed.

Few reports have described invasive meningococcal disease caused by nonserogroupable *N. meningitidis* strains; the lack of such reports suggests that complement deficiency might be a predisposing factor, and all the reported isolates were determined to be capsule null locus (cnl) strains, which lacked the genetic islands encoding the entire capsule (*5**–**7*). However, the patient described here was not found to have a complement deficiency, and the disease-associated nonserogroupable *N. meningitidis* strain in this study contained the genetic locus of an intact capsule.

In China, meningococcal polysaccharide vaccines A and C have been used for routine immunization. In many countries in Africa, repeated vaccination against *N. meningitidis* serogroups A and C have likely led to a selective increase in the incidence of meningococci of other serogroups, thereby resulting in a changed profile of meningococcal disease (*8*). In recent years, invasive disease caused by *N. meningitidis* serogroup W135 and serogroup X strains has emerged in China (*9**,**10*). Therefore, meningococcal disease caused by serogroups other than A and C as well as nonserogroupable *N. meningitidis* strains appears to be an emerging problem and should be investigated epidemiologically.

## References

[1] Shao Z, Li W, Ren J, Liang XF, Xu L, Diao BW, Identification of a new *Neisseria meningitidis* serogroup C clone from Anhui Province, China. Lancet. 2006;367:419–23. 10.1016/S0140-6736(06)68141-5 16458767

[2] Hammerschmidt S, Müller A, Sillmann H, Mühlenhoff M, Borrow R, Fox A, Capsule phase variation in *Neisseria meningitidis* serogroup B by slipped-strand mispairing in the polysialyltransferase gene (*siaD*): correlation with bacterial invasion and the outbreak of meningococcal disease. Mol Microbiol. 1996;20:1211–20. 10.1111/j.1365-2958.1996.tb02641.x 8809773

[3] Lavitola A, Bucci C, Salvatore P, Maresca G, Bruni CB, Alifano P. Intracistronic transcription termination in polysialyltransferase gene (*siaD*) affects phase variation in *Neisseria meningitidis.* Mol Microbiol. 1999;33:119–27. 10.1046/j.1365-2958.1999.01454.x 10411729

[4] Dolan-Livengood JM, Miller YK, Martin LE, Urwin R, Stephens DS. Genetic basis for nongroupable *Neisseria meningitidis.* J Infect Dis. 2003;187:1616–28. 10.1086/374740 12721942

[5] Vogel U, Claus H, von Müller L, Bunjes D, Elias J, Frosch M. Bacteremia in an immunocompromised patient caused by a commensal *Neisseria meningitidis* strain harboring the capsule null locus (cnl). J Clin Microbiol. 2004;42:2898–901. 10.1128/JCM.42.7.2898-2901.2004 15243035 PMC446252

[6] Hoang LM, Thomas E, Tyler S, Pollard AJ, Stephens G, Gustafson L, Rapid and fatal meningococcal disease due to a strain of *Neisseria meningitidis* containing the capsule null locus. Clin Infect Dis. 2005;40:e38–42. 10.1086/427875 15714405

[7] Findlow H, Vogel U, Mueller JE, Curry A, Njanpop-Lafourcade BM, Claus H, Three cases of invasive meningococcal disease caused by a capsule null locus strain circulating among healthy carriers in Burkina Faso. J Infect Dis. 2007;195:1071–7. 10.1086/512084 17330799

[8] Gagneux SP, Hodgson A, Smith TA, Wirth T, Ehrhard I, Morelli G, Prospective study of serogroup X *Neisseria meningitidis* outbreak in northern Ghana. J Infect Dis. 2002;185:618–26. 10.1086/339010 11865418

[9] Shao Z, Zhou H, Gao Y, Ren H, Xu L, Kan B, *Neisseria meningitidis* serogroup W135, China. Emerg Infect Dis. 2010;16:348–9. 20113581 10.3201/eid1602.090901 PMC2958009

[10] Chen C, Zhang TG, He JG, Wu J, Chen LJ, Liu JF, A first meningococcal meningitis case caused by serogroup X *Neisseria meningitidis* strains in China. Chin Med J (Engl). 2008;121:664–6. 18466690

